# The Utility and Experience with Disease Biomarkers in Juvenile Onset Arthritis vs. Adult Onset Arthritis

**DOI:** 10.7759/cureus.5131

**Published:** 2019-07-13

**Authors:** Anjali Patwardhan

**Affiliations:** 1 Child Health, University of Missouri, Columbia, USA

**Keywords:** biomarkers, juvenile idiopathic arthritis, rheumatoid arthritis, citrullinated protein/peptide antigens, rheumatoid factor, ana, erosive disease, american college of rheumatology, peptidyl arginine-deiminase enzyme

## Abstract

Juvenile idiopathic arthritis (JIA) is the most common but extremely heterogeneous group of rheumatic diseases of childhood. There are no reliable, well-researched and published biomarkers for diagnosis or monitoring in juvenile idiopathic arthritis as there are for rheumatoid arthritis (RA) in adults. Biomarkers are not utilized in classifying JIA as they are in adult RA, making the JIA classifications less clinically effective and informative. The situation presents a lost opportunity for early aggressive therapy in JIA patients. Various researchers have used diverse biomarkers anecdotally in JIA and more systematically in RA patients and have drawn inferences on their utility from their experiences. The experience with biomarkers from RA patients cannot necessarily be extrapolated for JIA patients because they are dissimilar diseases. This article reconnoiters the comparative utility of various arthritis biomarkers in adult as well as in JIA patients. In contrast to RA, JIA is in itself a diverse group of arthritis with clinically overlapping subgroups with diverse etiology. The difference in the etiopathogenesis of arthritis subgroups demands identifying subgroup-specific biomarkers for diagnosis/monitoring and subgroup-specific therapies for management. The diagnostic/prognostic value of the individual biomarker could be different in different types of arthritis and in different types of hosts. Understanding the utility of individual biomarkers and careful selection of the assay are important to achieve the best disease outcomes.

## Introduction and background

The most common rheumatic disease in childhood is juvenile idiopathic arthritis (JIA). European incidence rates ranged from 1.6-23/100,000 and prevalence rates from 3.8-400/100,000 while the average incidence and average prevalence rates in USA were 11.9/100,000 (95% CI 10.9-12.9) and 44.7 (95% CI 39.1-50.2), respectively, in 2009 [1]. The current trends indicate that prevalence and incidence rates are on the rise, which in part may be explained by growing awareness about the diseases and improving diagnostic tools. Juvenile idiopathic arthritis is heterogeneous multifactorial childhood arthritis. The oligo/polyarthritis subgroup of JIA for the most part is a disease of the adaptive immune system and mediated through autoreactive T-cells against cartilage antigens. The auto-antigens derived from the cartilage activate autoreactive Th1/ Th17 T-cells leading to secretion of IFN-γ and IL-17-pro-inflammatory cytokines, hence the inflammation. Also, there is inhibition of regulatory T (Treg) cells with reduced IL-10-anti-inflammatory cytokine, breaking the immune-tolerance for the self-antigens. In other words, the disproportion between autoreactive Th1/Th17 function vs. Treg cell function breaks the T-cell self-antigen-tolerance and leads to synovial inflammation. The other subtypes of JIA such as psoriatic arthritis, enthesitis-related arthritis, and systemic-onset JIA are all considered autoinflammatory diseases. The subtype systemic-onset-JIA (SOJIA) is a disease of the innate immune system. In SOJIA, there is anomalous activation of phagocytes (monocytes, macrophages, and neutrophils) with the abundant release of IL-1, IL-6, IL-18 and S100-proteins, which are pro-inflammatory cytokines and lead to systemic inflammation [2]. The difference in the etiopathogenesis of arthritis subgroups demands identification of different biomarkers for disease diagnosis/monitoring and different subgroup-specific therapies [3-4].

The anti-citrullinated protein antibodies are now included (2010) along with rheumatoid factor (RF) in the European League Against Rheumatism (EULAR) and American College of Rheumatology (ACR) diagnostic criteria for rheumatoid arthritis (RA) in adults.

The dilemma

For childhood arthritis, three different classifications are available from the American College of Rheumatology (ACR), the European League Against Rheumatism (EULAR), and the International League of Associations for Rheumatology (ILAR). All the three classifications are primarily based on clinical symptoms/signs within six months of onset. Unfortunately, sometimes the difference in the subtypes is clinically blurred. The classifications in childhood are not only varied but have limited prognostic value and do not consider biomarkers other than RF in the diagnosis and therefore limit the opportunities for early aggressive therapy for the patients. The RF test is positive only in a fraction of JIA patients as against most (75%) of the adult RA patients, which makes these JIA classifications even less informative. Research (Trial of Early Aggressive Therapy/ TREAT Trial) has shown that aggressive therapy is the key for both short and long-term outcomes in JIA patients (NCT00443430) [5].

An ideal biomarker should be disease-specific, should be able to detect the disease early in the course and must be positive in most patients with that disease, i.e., should have both, high sensitivity and specificity. Most of the biomarkers do not necessarily have a causal relationship with the disease [6]. Unfortunately, many biomarkers that are used in RA patients are not studied or researched in pediatric patients. The disease course in JIA is currently predicted by the age of onset, sex, race and some patterns in the course of the disease that physicians have learned through their own experience. Some of the clinical features used for prediction of aggressive disease course are as follows: bilaterally symmetrical involvement of joints, polyarthritis at onset, early age of onset, severe/extensive disease at onset, arthritis involving cervical spine, X-rays showing erosions or joint space narrowing, early involvement of hip and or wrist, ankle arthritis in the first year of onset, positive RF, thrombocytosis at six months of onset and/or persistence of systemic/extra-articular features in systemic-onset JIA [7-9]. Higher erythrocyte sedimentation rate (ESR) at onset is considered to be associated with an aggressive disease course in oligo JIA [10-11]. Inflammatory markers such as ESR and CRP are not only nonspecific and poorly sensitive for detecting disease but also do not closely mirror the JIA disease activity. Positive anti-nuclear antibodies (ANA) are considered a risk factor for uveitis in JIA. Some researchers believe that the manifestation of ankle arthritis within the first year of onset is a poor prognostic marker [9]. It is assumed in the classification of JIA that only polyarthritis patients need to be tested for rheumatoid factor (RF-positive and RF-negative polyarthritis).This assumption is not accurate. Another barrier for a comparative analysis of biomarkers is the poor standardization, inter and intra-assay variability, and non-availability of universally accepted laboratory biomarker testing tools across the world. This technical aspect of the biomarker assays makes a comparison of patient populations from different parts of the world difficult. RF is studied more frequently in JIA patients than anti-citrullinated protein/peptide antigens (ACPA). Some clinicians still believe that ACPA and RF are mutually exclusive tests [12-14].

## Review

Available biomarkers in arthritis

Family of Antibodies to Citrullinated Protein/Peptide Antigens (ACPA)

The human peptidyl arginine deiminase (PAD) enzyme has five isotypes expressed in several different parts of the body. This enzyme is responsible for citrullinating (replacing arginine residue with citrulline by deamination) the extracellular proteins of the dying cells. Citrullination is a normal physiologic process routinely occurring in the apoptotic cells. Out of the five isoforms of the PAD enzyme, type two and four (PAD2, PAD4) are expressed in inflammatory leukocytes and are released when these cells are dying. PAD4 is found in the cytoplasmic granules of eosinophils, the nucleus of neutrophils and synovial tissues.

Interestingly, some RA patients develop autoantibodies against enzyme PAD4 (anti-PAD4 antibodies), which are used as biomarkers in RA patients [15]. The anti-PAD4 antibodies can be detected several years before the onset of arthritis in RA patients. Some researchers believe that anti-PAD4 antibodies are also disease severity biomarkers and have functional significance in adult patients with RA. Most information on anti-PAD4 antibodies come from the patients who have established RA. The reported diagnostic sensitivity of anti-PAD4 antibodies ranges from 25% to 50%, while specificity ranges from (91%-99%). The anti-PAD4 antibodies are extremely rare in healthy adult controls and adult patients with other systemic autoimmune diseases. The occurrence and significance of anti-PAD4 autoantibodies in JIA or pediatric systemic autoimmune diseases is not known [15]. Citrullinated proteins have been demonstrated in the RA/JIA synovium by several researchers, but they are never identified in the synovium of a healthy person [16]. Noteworthily, RF can be positive in a small fraction of normal adults. Out of several, there are four extensively researched citrullinated antigens (fibrinogen, vimentin, collagen type II, and alpha-enolase) that are found in the synovium of the joints in arthritis patients. The inflammation is immune complex-mediated; citrullination changes the shape of the peptide/protein so that it is not recognized as ‘self’ by the immune system [17]. Therefore the immune system treats these citrullinated peptide/proteins as foreign antigens and starts producing antibodies against them. This family of diverse antibodies (IgG class) is called anti-citrullinated peptide antibodies (ACPA). Anti-perinuclear factor (APF) and anti-keratin antibodies (AKA) were the first two members of the ACPA family and were seen to recognize citrullinated epitopes of filaggrin peptide. ACPAs could be detected in adult RA patients several years before the onset of their clinical symptoms of RA [18-20]. ACPA family of antibodies are also reported as poor prognostic markers in adult RA patients for ischemic heart diseases/coronary artery disease (CAD) [21]. High burden of ACPAs is identified within atherosclerotic plaques. Some specific ACPAs such as citrullinated-fibrinogen are reported in the coronary plaques more often than other ACPAs. A causal relationship between citrullinated-fibrinogen and accelerated atherosclerosis in RA patients has been also reported [22].The citrullinated-fibrinogen is believed to be selectively bound by RA autoantibodies at a higher titer.

Anti-CCP Antibodies

Synonyms: CCP antibody; citrulline antibody; anti-citrulline antibody; anti-cyclic citrullinated peptide; anti-CCP; ACPA. Formal name: Cyclic citrullinated peptide antibody.

Three generations of the anti-cyclic citrullinated peptide (anti-CCP3) antibody assays evolved based on the citrullination theory. Lately, anti-Sa antibody, which is usually positive in Sjogren’s and Lupus patients, was added to the ACPAs family. The anti-Sa antibody is an antibody directed against citrullinated vimentin (also called anti-mutated citrullinated vimentin-anti-MCV antibody). The anti-MCV antibody has a similar but not the same diagnostic and prognostic performance when compared with RF and anti-CCP. It is specifically useful in the diagnosis of RF & anti-CCP negative RA patients. None of these antibodies are extensively researched in the pediatric population. Anti-CCP antibodies are more often but not exclusively positive in polyarticular patients, and their presence suggests severe erosive disease in both adults as well as in children [23]. A small group of RF-negative JIA, oligo-JIA, and systemic-onset-JIA (SOJIA) patients were also reported to be positive for anti-CCP antibodies. The above finding suggests that anti-CCP antibodies may have diagnostic value and a possible pathogenetic role even in SOJIA [24]. In a comparative study of 30 adult RA and 68 JIA patients, anti-CCP antibodies were reported to be more prevalent in RA than in JIA patients [23]. But this cannot be stated with full confidence because all these biomarkers are not routinely tested in JIA patients. No correlation has been established between positive antinuclear antibodies (ANA) and anti-CCP positivity in adult or pediatric patients [17, 23]. In a few studies, the anti-CCP test has been reported to have higher specificity but poor sensitivity in JIA as compared to adult RA patients [25-26]. The diagnostic sensitivity was higher in RF-positive polyarthritis than other subgroups of arthritis [25]. Lower sensitivity makes anti-CCP test a poor screening tool, but if positive in a patient with nonspecific arthritis or protracted arthralgias, it suggests an evolving seropositive/erosive arthritis.

In another comparative analysis of 59 JIA and 129 adult RA patients, the specificity and sensitivity of anti-CCP antibody were 99.1% and 10.2% in JIA and 98.4% and 55% in RA patients, respectively [26]. Researchers have reported that the disease duration and prior treatment did not correlate with anti-CCP positivity. A group of researchers believe that both anti-MCV and anti-CCP antibodies are especially useful in the early course of the disease as diagnostic markers. Anti-MCV antibody positivity is linked to radiologically destructive disease and can identify RF/anti-CCP negative patients who may need aggressive therapy [27].

The comparison amongst ACPA-isotypes biomarkers is difficult because of the poor test-standardization, poor inter-assay/intra-assay reliability of the various commercial assays and the absence of prospectively controlled clinical RCTs. Anti-CCP antibodies have modest false-positive rates (< 5%) as compared to RF (10% to 30%) [28].

To detect the citrullinated antibodies, various manufacturers use different citrullinated antigen substrates such as Epstein-Barr virus, IgG-derived peptides, synthetic cyclic peptides, mutated human vimentin, and recombinant rat filaggrin. Though tests from different manufacturers have little difference in their analytical methods such as titrating various sensitivities for a fixed specificity, they all are enzymatic immunoassay (EIA) tests. Research has shown that diagnostic accuracy and analytical imprecision can be influenced greatly by the antigenic source used in the test. The sensitivity and specificity of different manufacturers may vary. False-positive results are common in patients who had or were recovering from viral syndromes.

Anti-MCV Antibody

The new member of the ACPAs family of tests is genetically modified citrullinated vimentin (anti-MCV) assay. The anti-MCV antibody is an isoform of vimentin in which glycine is replaced by the arginine residues. Anti-MCV antibody’s diagnostic sensitivity is better than that of anti-CCP-2 antibodies, and its specificity is almost similar to anti-CCP-2 antibodies [29]. The anti-MCV antibody can also be positive in anti-CCP-negative and RF-negative patients; therefore, it can be used as a diagnostic test in those patients. Anti-MCV antibodies can increase the odds for the diagnosis in nonspecific-undifferentiated arthritis patients if used in conjunction with RF and anti-CCP tests. It can stratify seronegative arthritis patients with higher risk for erosive arthritis. In a systematic review (1966 to May 2008) the sensitivity of anti-MCV in the mixed population of nonspecific adult arthritis patients and established RA patients ranged from 0.64 to 0.84 and the specificity ranged from 0.79 to 0.96. The specificity and sensitivity range were comparable to anti-CCP in the same patient pools. The review also confirms the moderate association of anti-MCV positivity with radiological progression (Larsen score and Sharp-van der Heijde (SvH)), and the association strength was similar to that the anti-CCP antibodies had with the Larsen score and SvH score [28].

Though anti-MCV antibody is especially of diagnostic value in seronegative patients, double-positivity for anti-MCV and anti-CCP2 improves the specificity and positive predictive value in RA [30]. Importantly, as opposed to anti-CCP antibody titers, therapy with infliximab affects the anti-MCV antibody titers and clinical response significantly; therefore, it can be used to monitor the therapy [31]. The ACPAs family of antibodies are not specific to any JIA subtype and can be positive in any or all subtypes. Presence of more than one ACPA indicates more aggressive disease in JIA patients [32]. So far, there is no published information on the diagnostic utility of anti-MCV testing in the pediatric or adult arthritis patients who already have positive anti-CCP and RF testing. Anti-CCP and anti-MCV are not mutually exclusive tests, and patients can be positive for one but negative for another. Therefore, few researchers recommend testing anti-MCV in patients who are negative for one or both anti-CCP and/or RF tests, as a prognostic biomarker. It is not clear if anti-MCV (different citrullinated protein than anti-CCP) positive patients constitute a diverse disease subset that may progress to a diverse RA isotype. Hence, anti-MCV may have a dissimilar prognostic value than that of anti-CCP [33]. The ACPAs and class II MHC have a strong linkage. This linkage suggesting specificity of the peptides presented by HLA-DR to CD4+T, plays a significant role in the production of a specific ACPA through T cell activation [34]. The anti-CCP rather than anti-MCV antibodies in JIA are believed to suggest radiographic progression better [27].

RF vs. Anti-CCP Tests

A patient should have two RF positive tests, at least three months apart over a six months period to be consider RF positive. On the other hand, a single positive test is enough for the confirmation of positive anti-CCP antibody status. Few published studies have identified that about 6% of RF-negative patients are anti-CCP positive. Anti-CCP can also be present in oligoarthritis patients [24]. The RF and anti CCP positivity have a different significance from diagnosis as well as prognosis point of view and they are not mutually exclusive. There is strong opinion evolving amongst researchers that anti-CCP testing should be included in the classification of JIA also [35]. Polyarthritis patients constitute around 15%-25% of all JIA patients, and 15% of these polyarthritis patients are RF positive [36]. The prevalence of RF and anti-CCP in various JIA individual sub-groups is not known.

Rheumatoid Factor

Rheumatoid factor (RF) is the soluble antibodies (IgM) against self Fc-Fraction of immunoglobulins (IgA, IgG, IgM). In only 40% of RA, patients are positive for RF in their early years (first three months of disease onset), but approximately 75% of patients eventually become RF positive. RF should be tested in serum sample, which is not more than 14 days old (preferably refrigerated). The sample should be discarded in the presence of gross lipemia. The RF is measured by several different assays, i.e., egg radioimmunoassay (RIA), enzyme-linked immunoassay (ELISA), and agglutination of human IgG coated polystyrene latex particles, etc. Unfortunately, though all these assays have similar diagnostic/prognostic significance, there is no intra-assay/ inter-assay standardization that makes the results incomparable. Some researchers believe that in RA the isotypes of biomarkers do not add any further value from the diagnostic and prognostic point of view while others believe the contrary [37-38]. The RF is not considered causative of RA, its titers show a positive relationship with the severity of the disease, but they do not disappear with the drug-induced remissions. Occasionally the patient who is in drug-induced remission can have high titers of RF. The RF is not specific to RA but can be positive in several other diseases such as viral hepatitis, influenza, systemic lupus erythematosus (SLE), tuberculosis, polymyositis, syphilis, infectious mononucleosis, mixed connective tissue disease (MCTD), systemic sclerosis, JIA, etc. It is also observed in 1%-5% of healthy individuals. Due to poor positive predictive value as well as poor specificity, RF is considered positive only if it is positive in two out of three tests within six months. The antinuclear antibody called anti-RA33 is an ELISA assay (≥25 U/mL) and correlates with RF positivity though it can also be positive with all the other diseases as listed above. RA33 positive lupus patients run a more erosive disease course than those who are negative. As against RF, anti-RA33 antibody has an extremely wide range of diagnostic sensitivity (6%-75%) for RA. Anti-RA33 antibody is detected in the early course (first three months of onset) of the disease, and its titers fluctuate with disease activity. It gets barely positive i.e. the titers decrease at times of drug-induced-RA remissions. Because of anti-RA33 antibody’s wide range of diagnostic sensitivity, it cannot be used in the classification of RA. The discriminative capacity of anti-RA33 is (anti-RA33 test had 98% sensitivity, 20% specificity, 55% positive predictive value, and 90% negative predictive value) better and also complementary to RF [39].

Synovial Fluid Biomarkers

Synovial fluid investigations have been recommended as biomarkers to predict the disease course. Patients with mild vs. severe disease show different patterns in post-translational modifications of proteins and proteomes [40]. The difference in synovial fluid T-cell types and frequencies have not only been recognized as disease severity identifiers but also as an indicator of diverse disease etiopathogenesis [41]. Some researchers have shown that elevated chemokine CCL5 titer and low synovial fluid CD4:CD8 ratio is predictive of severe disease and if it is detected in oligoarticular patients, they are most likely to run an extended oligoarticular course [9, 42-44].

S100 Proteins

Several members of the S100 protein family in adults are identified as reliable predictors of erosive arthritis. Few calcium‐binding S100 proteins such as serum calgranulin-B (S100A9), calgranulin-A (S100A8), and calgranulin-C (S100A12) were found to be elevated in patients with erosive RA and not in milder RA patients or healthy adults. In systemic-onset JIA, S100 proteins are severely elevated and correlate with disease activity, relapses and can even be used to predict the flares [45]. Pro-inflammatory S100 proteins, S100A8/9 (AKA calprotectin, myeloid-related protein, MRP8/14) and S100A12 are considered sensitive indicators of disease severity, activity, and predictors for response to methotrexate therapy in patients with JIA [46]. In studies, the MRP8/14 level was found to be lower or normal in patients who responded to the methotrexate therapy vs. those who did not [44]. The patients who responded to methotrexate and lowered their MRP8/14 levels after treatment had higher MRP8/14 levels before the start of the therapy, higher joint counts, and higher ESR/CRP (i.e., higher diseases activity) vs. other groups [44]. The MRP8/14 can also be a useful marker of the subclinical disease activity which cannot be identified by clinical examination or other lab tests. Using MRP8/14 to guide when to stop the medication in a patient who is in clinical remission may help reduce the relapses in JIA patients. Unfortunately, the standardization of the S100 proteins assay is poor with vast intra-assay and inter-assay variability and labor-intensive process. The jury is out for a cluster test involving several members from the S100 protein family to be used as a flare or activity signature in SOJIA patients. Currently, several gene clusters such as RAB27a and SH2D1, etc. are being looked into as potential sources of mRNA-expression biomarkers that can differentiate JIA patients with or without macrophage activation syndrome [47].

Multiple Biomarker Score Cards

The multi-biomarker disease activity (MBDA) test has evolved based on the fact that using multiple biomarkers can improve sensitivity, specificity, and positive predictive value. The MBDA used in RA involves 12 biomarkers and 1-100 scores [47]. The MBDA tests are validated for RA and not JIA, but its use in the management of RA is still controversial. The MBDA response in the patients who are on IL-6 receptor (IL6R) inhibitors should be carefully interpreted because IL-6 is a contributor to the MBDA score. Therefore, patients on the IL-6 therapy show a smaller MBDA score response than reflected in DAS28-CRP score improvement [48].

The comparison between RF and anti CCP antibodies are detailed in Table 1.

**Table 1:** Comparison between RF and anti-CCP ACR - American College of Rheumatology, Anti-CCP - anti-cyclic citrullinated peptide, RA - rheumatoid arthritis, RF - rheumatoid factor, JRA - juvenile rheumatoid arthritis

Parameter	RF	Anti-CCP antibodies
Can be used for early diagnosis	No	Yes
Can predate the first symptoms by several months or years	No	Yes
Can be found in healthy individuals	Yes	No
Has a high positive predictive value for the erosive joint disease if it is positive	Yes	Yes: better than RF
Specificity	~85%	~95%
Sensitivity	~ 69%	~ 67%
Can be positive in patients with hepatitis-C virus (HCV)-associated cryoglobulinemia	Yes	No
Can be positive in undifferentiated polyarthritis	Yes. RF-positive undifferentiated arthritis may or may never progress to RA/JRA	Yes. The anti-CCP positive undifferentiated arthritis will certainly progress to RA/JRA
It is a part of ACR diagnostic criteria for RA	Yes	Yes: recently added
The titer of the antibodies decreases with treatment	Yes	Yes
Differential response to anti-TNF therapy (Differential response of the rheumatoid factor and anti-citrullinated protein antibodies during adalimumab infliximab treatment in patients with rheumatoid arthritis suggests that RF and anti-CCP antibodies are independent autoantibody systems)	Yes	Yes
The titers decrease when treated with infliximab	Yes	No
Clinical response to anti-TNF	RF-positive patients clinically respond well	Anti-CCP positive patients clinically respond not so well
Can be used as an early predictor of the efficacy of anti-TNF therapy	Yes	Yes
HLA-DRB1 alleles carrying the shared epitope (SE) association	No	Yes
Correlation with Th1/Th2 cytokines	Yes	No
Positive in other autoimmune conditions, such as lupus, Graves’ disease and Sjogren syndrome	Yes	No
Positive in tuberculosis	No	Maybe
Levels of autoantibody may fluctuate over time but will not go away	Yes	Yes
Recommended as a screening test	Yes	No

Serum IL-6 Titer

IL-6 titers have been studied in RA but not in oligo/polyarticular subtypes of JIA. A high concentration of IL-6 is often detected in the synovial fluid in patients with active RA. It was hypothesized that plasma IL-6 concentrations might better reflect synovial inflammation than acute-phase reactants. In one study of 51 RA patients early in their disease course, the IL6 levels correlated with the acute phase reactant levels but there was no correlation identified between IL-6 levels and radiological damage [1].

ANCA and p-ANCA antibodies 

Antineutrophil cytoplasmic antibody (ANCA) and atypical (non-myeloperoxidase) p-ANCA can be positive in RA patients and rarely in JIA, but they have no diagnostic or prognostic significance [49].

## Conclusions

The diagnostic, prognostic, and predictive therapeutic response studies based on biomarkers is extensive in patients with RA as compared to those with JIA. The biomarkers are not yet used to diagnose or classify arthritis in JIA as in RA patients. The interpretations of the biomarkers from adult RA patients cannot just be extrapolated and used in childhood arthritis because these two are not the same disease. In contrast to RA, JIA is in itself a diverse group of arthritis with clinically overlapping subgroups and with diverse etiology. The diverse etiopathogenesis in JIA subgroups may have individual subgroup-specific biomarkers, which is yet to be explored. Biomarkers could be the ultimate tool for precise diagnosis, predicting disease course, being able to provide early aggressive therapy, and minimizing the disease damage. Prospective, randomized controlled multicenter research in JIA subtypes-biomarkers demands urgent attention. The key takeaway is that the diagnostic/prognostic value of the individual type of ACPA in diverse JIA subgroups may be different. Therefore, careful selection of the assay is critical for the meaningful use of the biomarkers to achieve best disease outcomes.
